# Supplementary material for: Autophagy disruption reduces mTORC1 activation leading to retinal ganglion cell neurodegeneration associated with glaucoma
Source: bioRxiv. 2023 Jan 4:2023.01.04.522687. Preprint. [Version 1] doi: 10.1101/2023.01.04.522687 (PMC9881969; doi:10.1101/2023.01.04.522687)
Supplement: 1 [file NIHPP2023.01.04.522687V1-supplement-1.pdf]

bioRxiv preprint doi: https://doi.org/10.1101/2023.01.04.522687; this version posted January 7, 2023. The copyright holder for this preprint (which was not certified by peer review) is the author/funder, who has granted bioRxiv a license to display the preprint in perpetuity. It is made available under aCC-BY 4.0 International license.

Supporting information

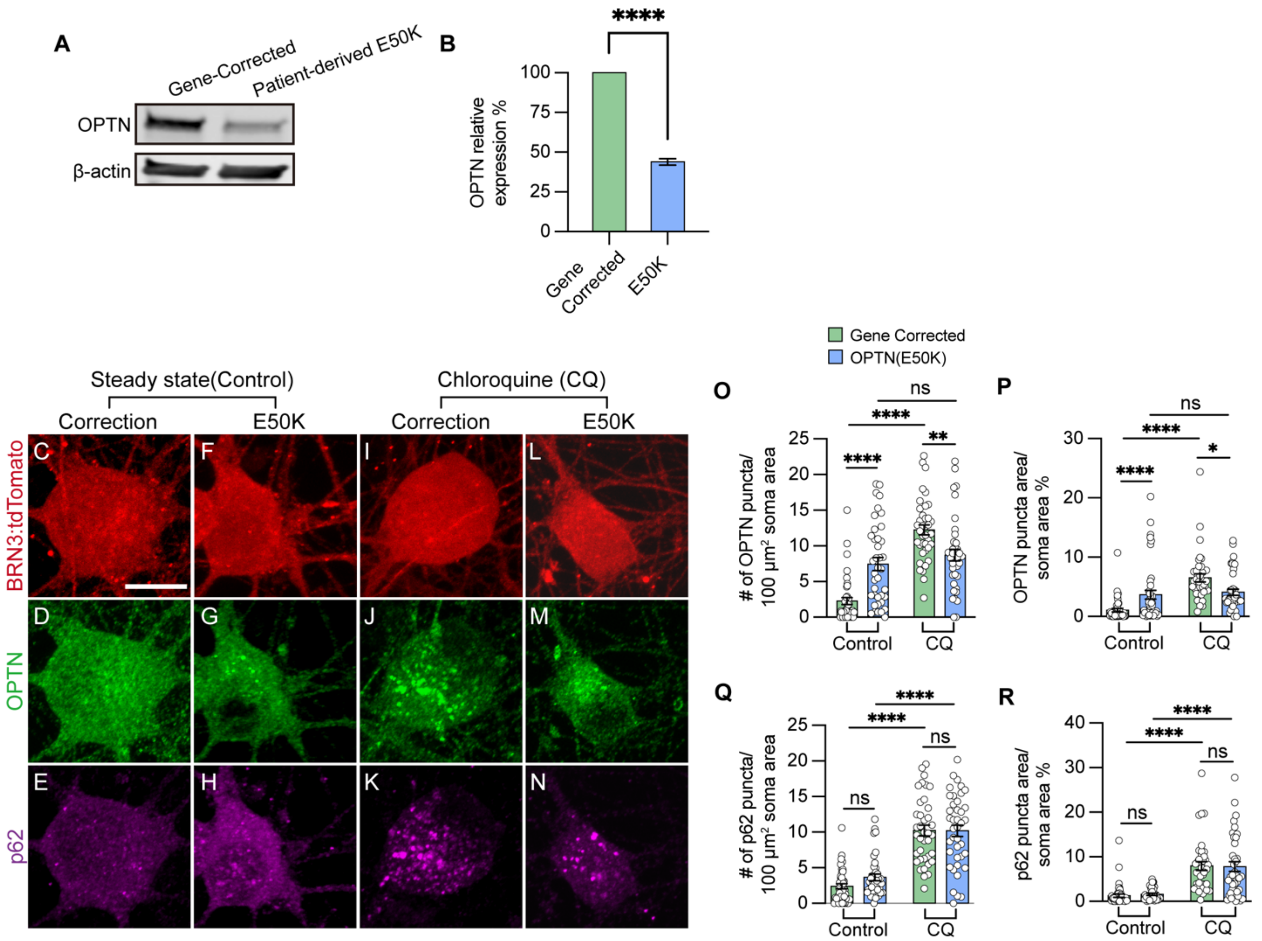

Supplemental Figure 1. Confirmation of changes in OPTN levels in RGC-derived from patient-specific iPSCs with OPTN(E50K) mutation compared to corresponding isogenic control line. (A-B) Western blot of OPTN relative to β-actin in patient-derived OPTN(E50K) and gene-corrected isogenic control iPSC-RGCs (n=5 for each WT and E50K; t-test, ****p<0.0001). (C-N) Immunostaining revealed the aggregation of OPTN and p62 puncta in BRN3:tdTomato iPSC-RGCs in patient-derived OPTN(E50K) mutant and gene-corrected isogenic control lines after steady state (control) (C-H) and chloroquine (CQ) treatment (I-N). Scale bar: 10 µm. (O-R) Quantification of OPTN puncta (O and P) or p62 puncta (Q and R) in iPSC-RGC (n=3 biological replicates using Ctrl-WT n=42, Ctrl-E50K n=39, CQ-WT n=38 and CQ-E50K n=40 technical

bioRxiv preprint doi: https://doi.org/10.1101/2023.01.04.522687; this version posted January 7, 2023. The copyright holder for this preprint (which was not certified by peer review) is the author/funder, who has granted bioRxiv a license to display the preprint in perpetuity. It is made available under aCC-BY 4.0 International license.

replicates; One-way ANOVA, Tukey post hoc test. ****p<0.0001, **p<0.01, *p<0.05, ns= not significant). Data are all represented as mean values ± S.E.M.

Supplemental figure 1-source data 1. The alternation of OPTN protein in gene corrected and patient derived-OPTN(E50K) mutation hPSC-RGCs.

bioRxiv preprint doi: https://doi.org/10.1101/2023.01.04.522687; this version posted January 7, 2023. The copyright holder for this preprint (which was not certified by peer review) is the author/funder, who has granted bioRxiv a license to display the preprint in perpetuity. It is made available under aCC-BY 4.0 International license.

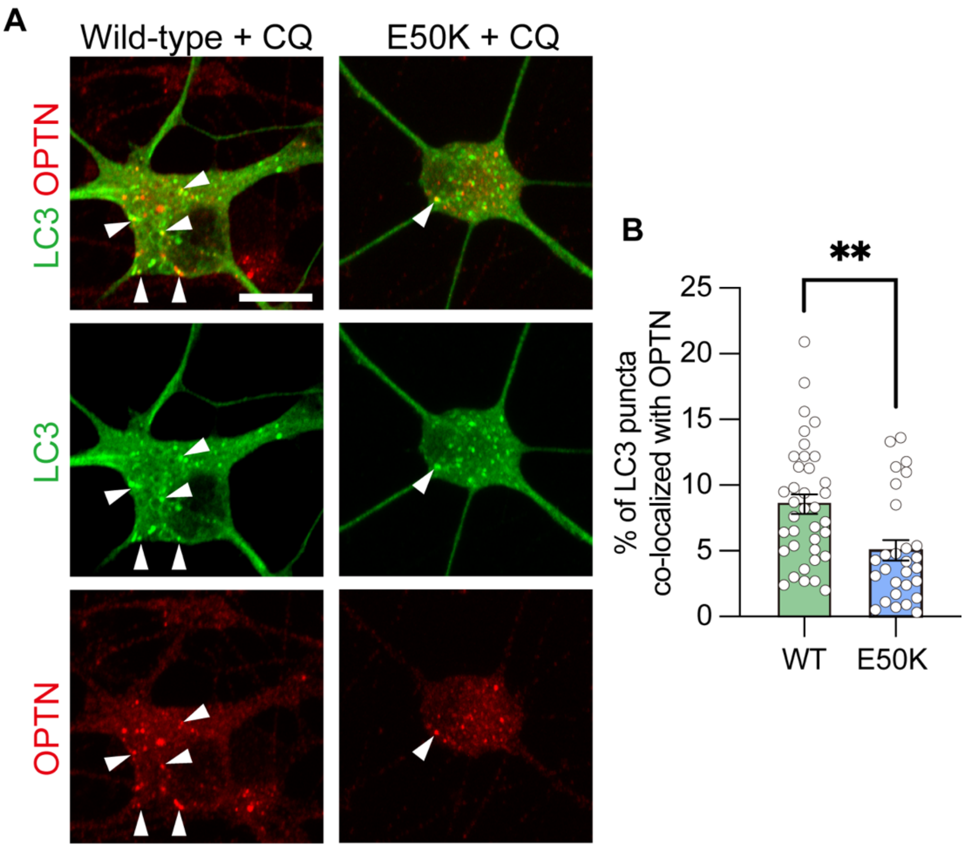

Supplemental Figure 2. Confirmation of reduced recruitment of LC3 by OPTN in patient-derived OPTN(E50K) iPSC-RGCs after chloroquine treatment. (A) Representative images of OPTN and LC3 localization in patient-derived iPSC-RGCs from wild-type and OPTN(E50K) cell lines after chloroquine treatment. White arrows identify puncta colocalized with OPTN and LC3. (B) Quantification of colocalization between OPTN and LC3 in patient-derived iPSC-RGCs (n=3 biological replicates using WT n=37 and E50K n=28 technical replicates; t-test, **p>0.005). Scale bar: 10 µm. Data are all represented as mean values ± S.E.M.

bioRxiv preprint doi: https://doi.org/10.1101/2023.01.04.522687; this version posted January 7, 2023. The copyright holder for this preprint (which was not certified by peer review) is the author/funder, who has granted bioRxiv a license to display the preprint in perpetuity. It is made available under aCC-BY 4.0 International license.

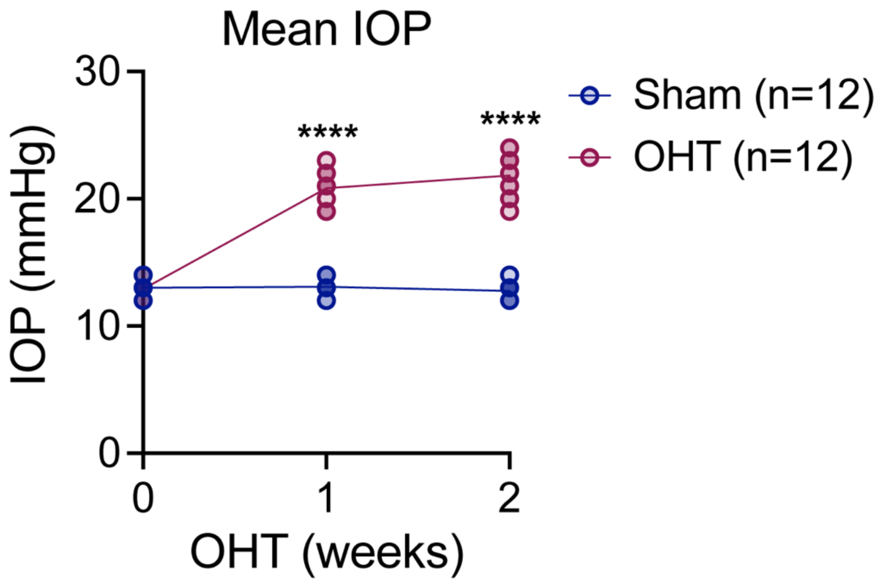

Supplemental Figure 3. Elevation of intraocular pressure in an ocular hypertension glaucoma model. Maintained elevation of intraocular pressure in mouse eyes following the injection of magnetic microbeads into the mouse anterior chamber, compared to sham-injected controls. Two-way ANOVA with Tukey's multiple comparison pos hoc test, ****$p < 0.0001$, $n = 12$ mice/group.

bioRxiv preprint doi: https://doi.org/10.1101/2023.01.04.522687; this version posted January 7, 2023. The copyright holder for this preprint (which was not certified by peer review) is the author/funder, who has granted bioRxiv a license to display the preprint in perpetuity. It is made available under aCC-BY 4.0 International license.

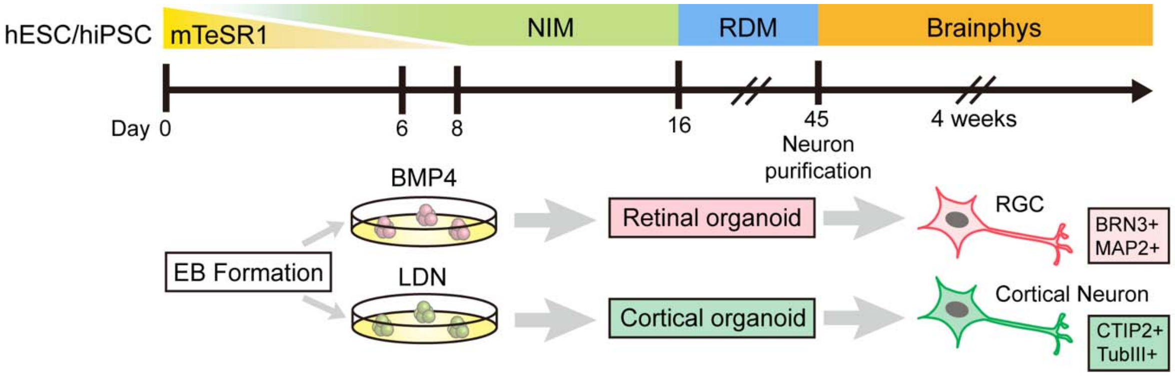

Supplemental Figure 4. Schematic diagram outlining the differentiation of RGCs and cortical neurons from hPSCs.

bioRxiv preprint doi: https://doi.org/10.1101/2023.01.04.522687; this version posted January 7, 2023. The copyright holder for this preprint (which was not certified by peer review) is the author/funder, who has granted bioRxiv a license to display the preprint in perpetuity. It is made available under aCC-BY 4.0 International license.

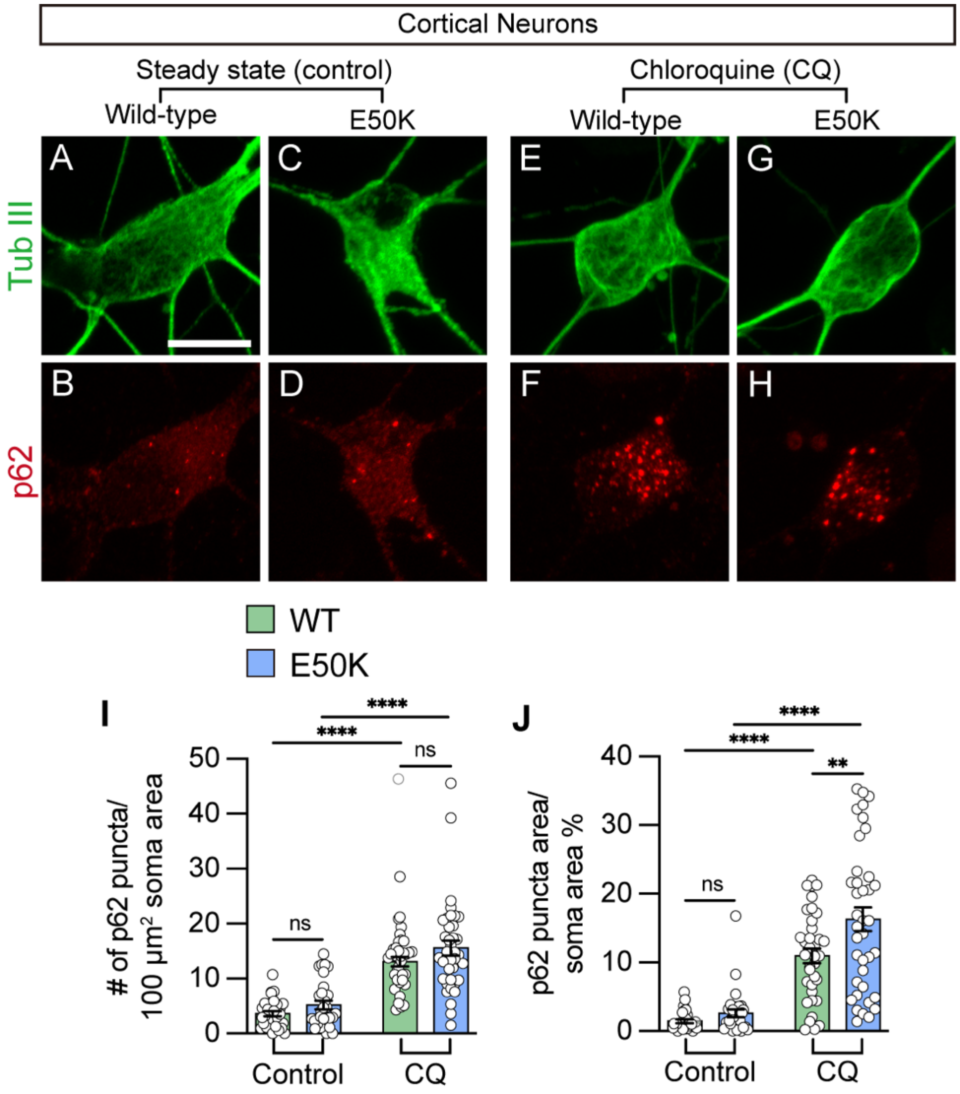

Supplemental Figure 5. Characterization of p62 expression in hPSC-cortical neurons from wild-type and OPTN(E50K) cell lines. (A-H) Representative images of p62 puncta in iPSC-derived cortical neurons from WT and OPTN(E50K) cell lines under steady state (control) (A-D) and chloroquine (CQ) treatment (E-H). Scale bar: 10 µm. (I-J) Quantification of p62 puncta in iPSC-derived cortical neurons (n=3 biological replicates using Ctrl-WT n=30, Ctrl-E50K n=30, CQ-WT n=36 and CQ-E50K n=38 technical replicates; One-way ANOVA, Tukey post hoc test. ****$p<0.0001$, **$p<0.01$, ns= not significant, $p>0.05$). Data are all represented as mean values ± S.E.M.

bioRxiv preprint doi: https://doi.org/10.1101/2023.01.04.522687; this version posted January 7, 2023. The copyright holder for this preprint (which was not certified by peer review) is the author/funder, who has granted bioRxiv a license to display the preprint in perpetuity. It is made available under aCC-BY 4.0 International license.

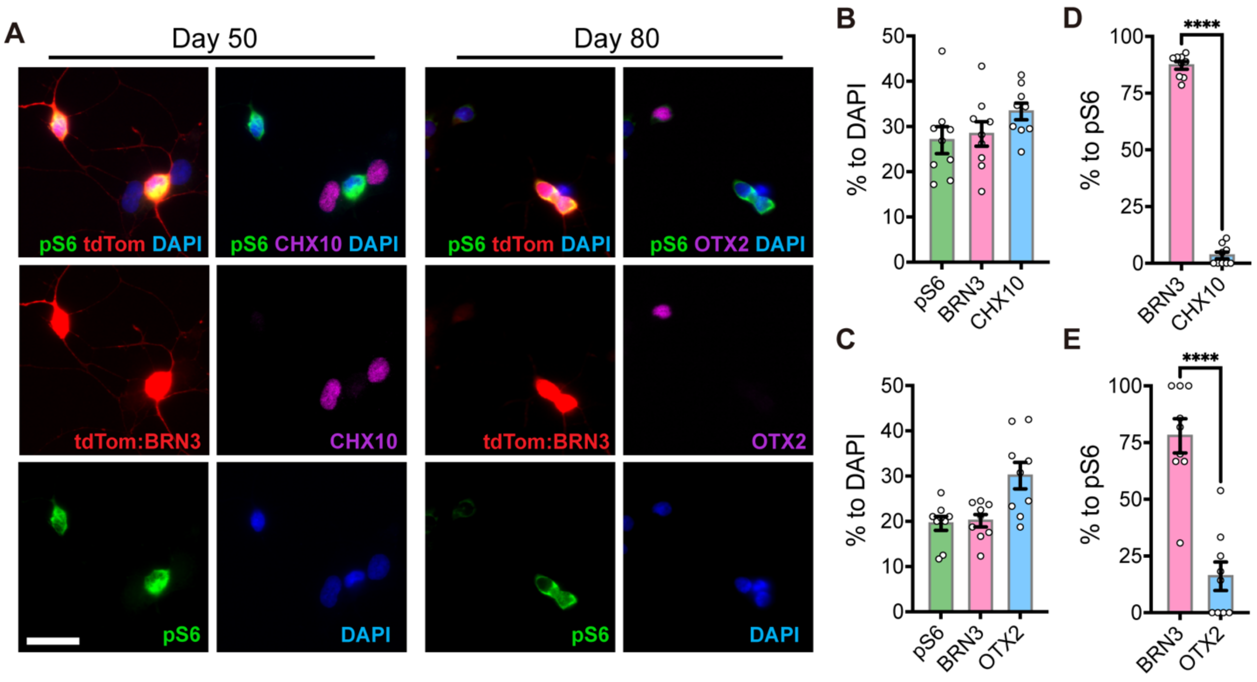

Supplemental Figure 6. mTORC1 activity is preferentially observed within RGCs among hPSC-derived retinal cells. (A) Retinal organoids were dissociated and plated onto laminin-coated coverslips at either day 50 or day 80 of differentiation to acquire the majority of major retinal cell types, including RGCs (BRN3:tdTomato), retinal progenitor cells (CHX10), and photoreceptors (OTX2), following by analysis of mTORC1 activity based upon co-staining with pS6Ser240/244. Scale bar: 25 µm. (B-C) Quantification of results showing the percentage of retinal cell types observed at day 50 or day 80 (n=9 images from three technical replicates). (D-E) Quantification of pS6Ser240/244 expression colocalized with either BRN3B:tdTomato, CHX10 or OTX2, suggesting that mTORC1 signaling is highly expressed in RGCs, with little expression in retinal progenitor cells or photoreceptors (n=9 images from three technical replicates; t-test, BRN3 vs CHX10: $p<0.0001$, BRN3 vs OTX2: $p<0.0001$). Data are all represented as mean values ± S.E.M.

bioRxiv preprint doi: https://doi.org/10.1101/2023.01.04.522687; this version posted January 7, 2023. The copyright holder for this preprint (which was not certified by peer review) is the author/funder, who has granted bioRxiv a license to display the preprint in perpetuity. It is made available under aCC-BY 4.0 International license.

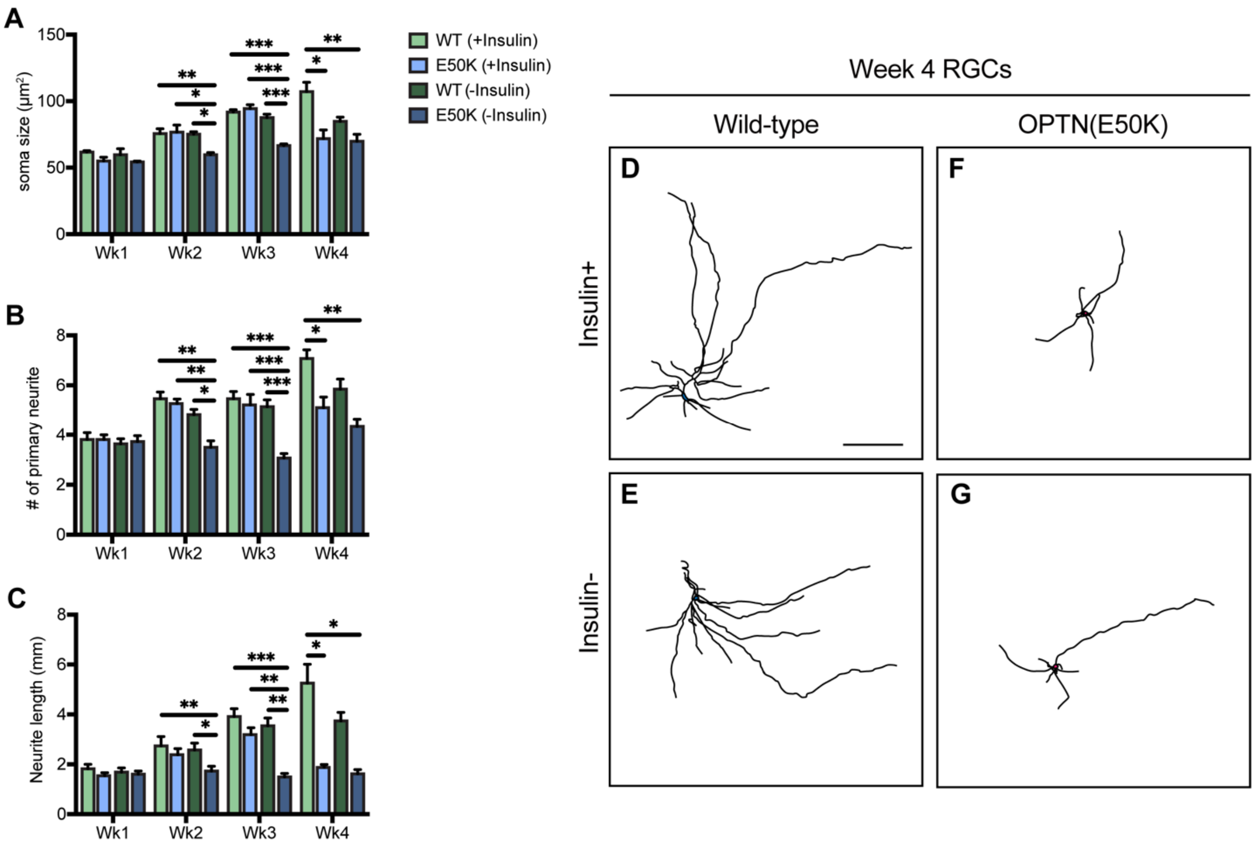

Supplemental Figure 7. Insulin deprivation expedites the onset of degenerative phenotypes in hPSC-RGCs with the OPTN(E50K) mutation. (A-C) Quantitative analysis of neurite measurements over the course of 4 weeks of differentiation in wild-type and OPTN(E50K) hPSC-RGCs grown with or without insulin, as measured by soma size (n≥30 each condition with 4 biological replicates; One-way ANOVA, Tukey post hoc test. ***p<0.001, **p<0.01, *p<0.05) (A), number of primary neurites (n≥10 each condition with 4 biological replicates; One-way ANOVA, Tukey post hoc test. ***p<0.001, **p<0.01, *p<0.05) (B), total neurite length (n≥10 each condition with 4 biological replicates; One-way ANOVA, Tukey post hoc test. ***p<0.001, **p<0.01, *p<0.05) (C). Data are all represented as mean values ± S.E.M. (D-G) Representative neurite tracings of WT and OPTN(E50K) hPSC-RGCs after 4 weeks of growth either with or without insulin. Scale bar: 200 µm.

bioRxiv preprint doi: https://doi.org/10.1101/2023.01.04.522687; this version posted January 7, 2023. The copyright holder for this preprint (which was not certified by peer review) is the author/funder, who has granted bioRxiv a license to display the preprint in perpetuity. It is made available under aCC-BY 4.0 International license.

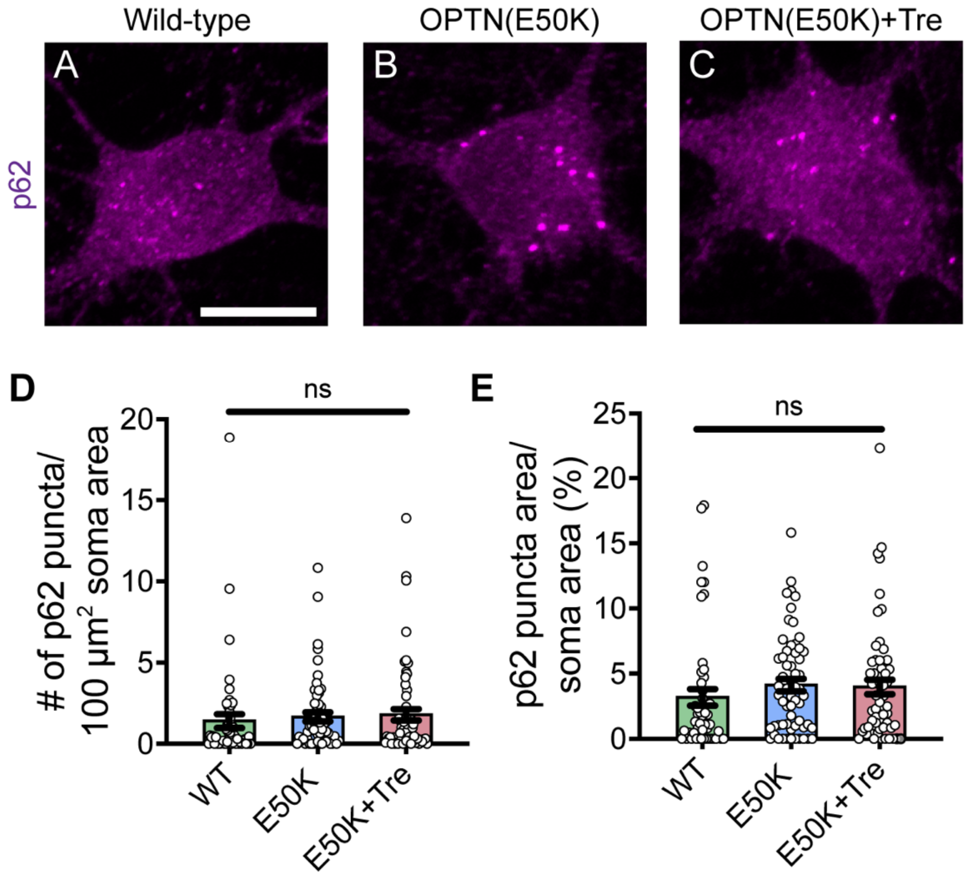

Supplemental Figure 8. p62 expression remains unchanged in hPSC-RGCs comparing wild-type, OPTN(E50K) and OPTN(E50K) plus trehalose conditions. (A-C) Representative images of p62 puncta in hPSC-RGCs. Scale bar: 10 μm. **(**D-E) Quantification of p62 puncta in hPSC-RGCs (n=3 biological replicates using WT n=51, E50K n=60, and E50K-trehalose n=61 technical replicates; One-way ANOVA, Tukey post hoc test. ns= not significant, p>0.05). Data are all represented as mean values ± S.E.M.

bioRxiv preprint doi: https://doi.org/10.1101/2023.01.04.522687; this version posted January 7, 2023. The copyright holder for this preprint (which was not certified by peer review) is the author/funder, who has granted bioRxiv a license to display the preprint in perpetuity. It is made available under aCC-BY 4.0 International license.

Supplemental Table. List of antibodies.

Antibody	Type	Source	Catalog	RRID	WB dilution	IF dilution
AKT	Mouse monoclonal	Cell Signaling Technology	2920	AB_1147620	1:2000	
AMPK	Rabbit polyclonal	Cell Signaling Technology	2532	AB_330331	1:1000	
β-actin	Mouse monoclonal	Sigma	A5441	AB_476744	1:10000	
BRN3	Goat polyclonal	Santa Cruz	SC-6026	AB_673441		1:200
CHX10	Goat polyclonal	Santa Cruz	SC-21690	AB_2216006		1:200
CTIP2	Rat monoclonal	Abcam	Ab18465	AB_2064130		1:500
LAMP1	Rabbit monoclonal	Cell signaling Technology	9091	AB_2687579	1:1000	
LAMP1	Rat monoclonal	DSHB	1D4B	AB_2134500	1:200	1:20
LC3 A/B	Rabbit monoclonal	Cell Signaling Technology	12741	AB_2617131	1:1000	
MAP1LC3A	Rabbit monoclonal	Abcam	ab185036	AB_881226		1:200
MAP2	Mouse monoclonal	Synaptic Systems	188011	AB_11042001		1:200
OPTN	Rabbit polyclonal	Novus	NBP1-84682	AB_11032496	1:1000	1:200
OTX2	Goat polyclonal	R&D Systems	AF1979	AB_2157172		1:2000
p62	Mouse monoclonal	Abcam	ab56416	AB_945626	1:2000	1:50
pAKT	Rabbit monoclonal	Cell Signaling Technology	4060	AB_2315049	1:2000	
pAMPK	Rabbit monoclonal	Cell Signaling Technology	2535	AB_331250	1:1000	
pS6	Rabbit polyclonal	Cell Signaling Technology	2215	AB_331682	1:1000	1:200
RBPMS	Guinea pig polyclonal	PhosphoSolutions	1832-RBPMS	AB_2492226		1:500
RFP	Rabbit polyclonal	Rockland	600-401-379	AB_2209751		1:200
RFP	Goat polyclonal	Origene	AB1140-100	AB_2877097		1:200
RFP	Mouse monoclonal	Rockland	200-301-379	AB_2611063		1:200
S6	Rabbit monoclonal	Cell Signaling Technology	2217	AB_331355	1:1000	

bioRxiv preprint doi: https://doi.org/10.1101/2023.01.04.522687; this version posted January 7, 2023. The copyright holder for this preprint (which was not certified by peer review) is the author/funder, who has granted bioRxiv a license to display the preprint in perpetuity. It is made available under aCC-BY 4.0 International license.

Tubulin,β-III	Rabbit polyclonal	Biolegend	802001	AB_2564645		1:500
